# Cryo-EM Structures of the Klebsiella pneumoniae AcrB Multidrug Efflux Pump

**DOI:** 10.1128/mbio.00659-23

**Published:** 2023-04-17

**Authors:** Zhemin Zhang, Christopher E. Morgan, Robert A. Bonomo, Edward W. Yu

**Affiliations:** a Department of Pharmacology, Case Western Reserve University School of Medicine, Cleveland, Ohio, USA; b Louis Stokes Cleveland Department of Veterans Affairs Medical Center, Cleveland, Ohio, USA; MedImmune

**Keywords:** antimicrobial resistance, cryo-EM, *Klebsiella pneumoniae* AcrB, multidrug efflux pump

## Abstract

The continued challenges of the COVID-19 pandemic combined with the growing problem of antimicrobial-resistant bacterial infections has severely impacted global health. Specifically, the Gram-negative pathogen Klebsiella pneumoniae is one of the most prevalent causes of secondary bacterial infection in COVID-19 patients, with approximately an 83% mortality rate observed among COVID-19 patients with these bacterial coinfections. K. pneumoniae belongs to the ESKAPE group of pathogens, a group that commonly gives rise to severe infections that are often life-threatening. Recently, K. pneumoniae carbapenemase (KPC)-producing K. pneumoniae has drawn wide public attention, as the mortality rate for this infection can be as high as 71%. The most predominant and clinically important multidrug efflux system in K. pneumoniae is the acriflavine resistance B (AcrB) multidrug efflux pump. This pump mediates resistance to different classes of structurally diverse antimicrobial agents, including quinolones, β-lactams, tetracyclines, macrolides, aminoglycosides, and chloramphenicol. We here report single-particle cryo-electron microscopy (cryo-EM) structures of K. pneumoniae AcrB, in both the absence and the presence of the antibiotic erythromycin. These structures allow us to elucidate specific pump-drug interactions and pinpoint exactly how this pump recognizes antibiotics.

## INTRODUCTION

Pneumonia is a serious lung infection that can be fatal if not efficiently resolved. In 2019, 2.5 million deaths were attributed to this disease (1). It can be especially devastating for young children with still developing immune systems, as almost a third of these victims were under 5 years old. In fact, pneumonia was the cause of 16% of all deaths of children in this age range (2). The Gram-negative pathogen Klebsiella pneumoniae is an opportunistic bacterium that is able to initiate different types of health care-associated infections, including pneumonia, septicemia, wound or surgical site infections, and meningitis (3). Unfortunately, K. pneumoniae has developed antimicrobial resistance and has become an immediate threat to our global health. A recent study of K. pneumoniae isolates from intensive care units in the northern region of Brazil indicated that most of these isolates have a high level of resistance to carbapenems, aminoglycosides, quinolones, tigecycline, and the last resort drug colistin (4). If current trends continue, infections caused by K. pneumoniae may become untreatable within the next decade. The continued COVID-19 pandemic has severely challenged our global health care worldwide. This is exacerbated when combined with the growing problem of bacterial antimicrobial resistance. It appears that K. pneumoniae and Acinetobacter baumannii are the most prevalent causative agents for secondary bacterial infection in COVID-19 patients, as high incidence of secondary infection caused by these two Gram-negative pathogens was observed in patients with severe COVID-19 (5). Owing to the lack of knowledge of these coinfections, the choice of treatments with respect to antibiotic therapies in these patients were made with very limited clinical experience and a paucity of scientific evidence (6). It is worth noting that an alarming mortality rate of 83% among those with COVID-19-bacterial coinfections has been reported in comparison with an overall mortality rate of 38.1% of total hospital-admitted COVID-19 patients (5).

K. pneumoniae belongs to the ESKAPE (Enterococcus faecium, Staphylococcus aureus, K. pneumoniae, A. baumannii, Pseudomonas aeruginosa, and Enterobacter species) group of pathogens. They are the leading cause of nosocomial infections and present one of the greatest challenges in modern medicine, as most of these bacteria have evolved to become resistant to multiple antimicrobials (7). ESKAPE bacteria commonly cause life-threatening infections, particularly in those that are critically ill and/or immunocompromised. Recently, K. pneumoniae carbapenemase (KPC)-producing K. pneumoniae has drawn wide attention for its pathogenesis, as the mortality rate for this infection can be as high as 71% (8). Due to its severe threat to human health, the World Health Organization (WHO) has listed carbapenem-resistant *Enterobacteriaceae*, including K. pneumoniae, as first-priority pathogens for the research and development of new antibiotics (9).

Multidrug efflux is considered to be one of the major causes of failure of drug-based treatments of infectious diseases (10). In K. pneumoniae, the best characterized multidrug efflux system is the prevalent acriflavine resistance B (AcrB) multidrug efflux pump (11–14). This efflux system is capable of mediating resistance to a broad spectrum of clinically relevant antimicrobial agents, including quinolones, β-lactams, tetracyclines, macrolides, aminoglycosides, and chloramphenicol (14). The K. pneumoniae AcrB (*Kp*AcrB) multidrug efflux pump belongs to the resistance-nodulation-cell division (RND) superfamily of efflux transporters (15). This inner membrane efflux pump assembles with the *Kp*AcrA periplasmic adaptor protein and the *Kp*TolC outer membrane channel to form a tripartite efflux complex that spans the entire cell envelope to directly extrude drugs out of K. pneumoniae cells (14). This drug efflux process is dependent upon the proton-motive force (PMF), which provides energy to the K. pneumoniae AcrAB-TolC system to actively export drug molecules (14).

To understand the mechanisms of drug recognition and extrusion of the *Kp*AcrB multidrug efflux pump, we here define cryo-electron microscopy (cryo-EM) structures of this membrane protein embedded in lipidic nanodiscs, in both the absence and the presence of the antibiotic erythromycin (Ery). We also use computational docking to predict how *Kp*AcrB recognizes a variety of antibiotics. The structural information allows us to elucidate specific pump-Ery interactions and determine how this pump binds the Ery antibiotic.

## RESULTS

### Structure of K. pneumoniae AcrB in the absence of erythromycin.

**FIG 1:**
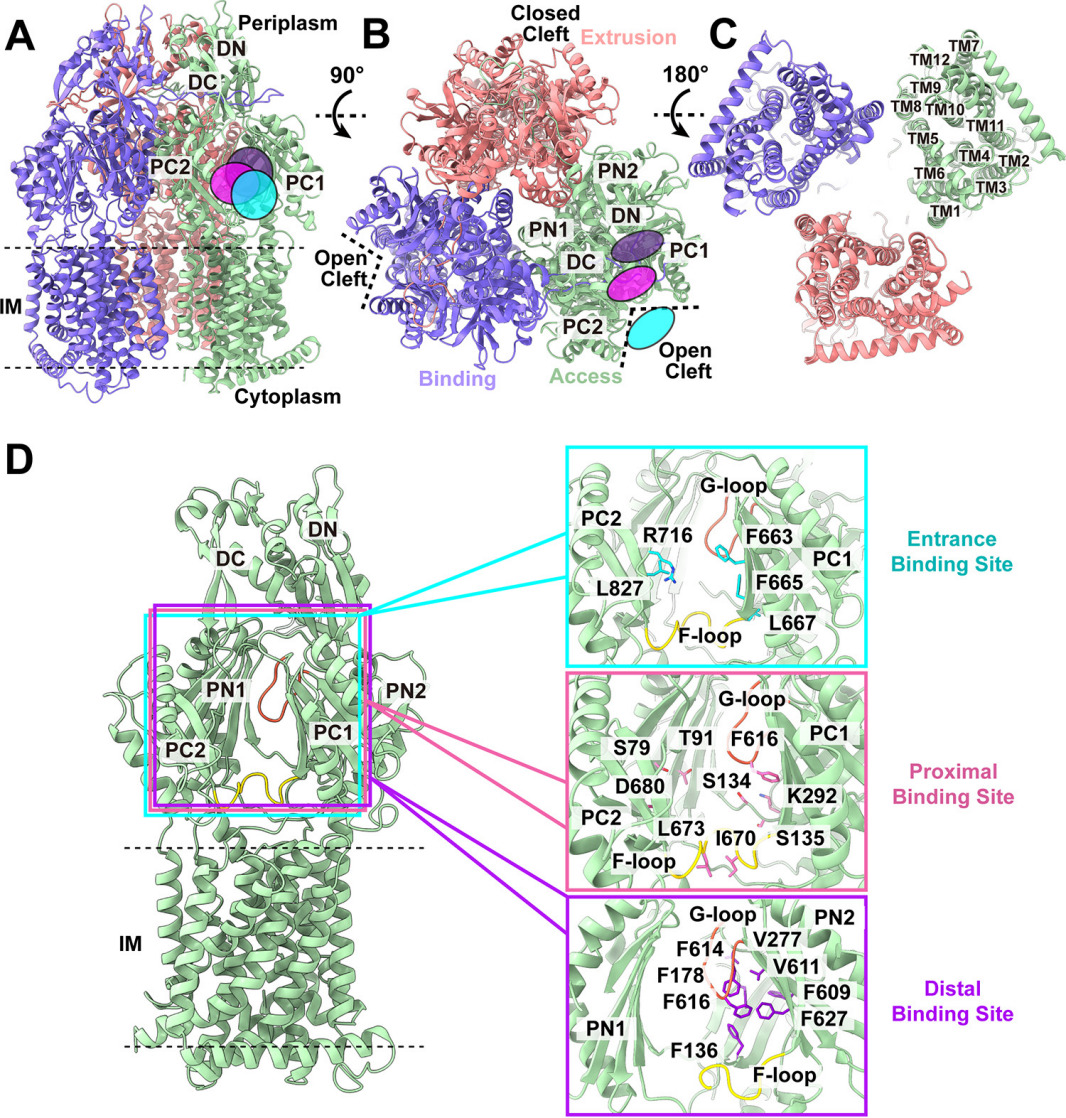
Cryo-electron microscopy (Cryo-EM) structure of *Kp*AcrB. (A to C) Ribbon diagrams of the structures of the side view (viewed in the membrane plane) (A), top view (viewed from the extracellular space) (B), and bottom view (viewed from the cytoplasm) (C) of the *Kp*AcrB trimer. In panels A to C, the “extrusion,” “access,” and “binding” protomers are colored pink, green, and slate, respectively. Each protomer of *Kp*AcrB contains 12 transmembrane helices (TM1 to TM12) and six periplasmic subdomains (PN1, PN2, PC1, PC2, DN, and DC). The locations of the entrance, proximal, and distal drug-binding sites are indicated with cyan, pink, and purple, respectively. (D) The entrance, proximal, and distal drug-binding sites. A “binding” protomer of *Kp*AcrB is included to indicate the locations of these binding sites with respect to the full-length protomer. The secondary structural elements of the “binding” protomer of *Kp*AcrB are colored green. The flexible loop (F-loop) and the gate loop (G-loop) are colored yellow and orange. Residues predicted to contribute to form these drug-binding sites are highlighted with sticks (cyan, entrance; pink, proximal; dark violet, distal). IM, inner membrane.

We cloned the full-length *Kp*AcrB multidrug efflux pump, which contains a 6×His tag at the N terminus, into pET15b to generate the pET15bΩ*KpacrB* expression vector. The *Kp*AcrB membrane protein was overproduced from E. coli BL21(DE3)Δ*acrB* cells and purified using a Ni^2+^-affinity column. Of note, *Kp*AcrB shares a 91% amino acid identity with that of E. coli AcrB (*Ec*AcrB), which facilitates a direct comparison between the two inner membrane proteins. Purified *Kp*AcrB was then reconstituted into lipidic nanodiscs, and its structure was determined using single-particle cryo-electron microscopy (cryo-EM) (Fig. 1; Fig. S1). The three-dimensional reconstitution of *Kp*AcrB led to a cryo-EM map at a nominal resolution of 2.82 Å (Fig. S1; Table S1), enabling us to build a model of this pump. The full-length *Kp*AcrB protein consists of 1,048 amino acids. Among these residues, we obtained very good quality cryo-EM densities from the first 1,033 residues. Therefore, our final structural model includes residues 1 to 1,033 of *Kp*AcrB.

10.1128/mbio.00659-23.1 FIG S1 Apo-*Kp*AcrB Data processing. (A) Data processing workflow of apo-*Kp*AcrB. Side and top views of the apo-*Kp*AcrB density maps are shown. (B) Representative two-dimensional (2D) classes apo-*Kp*AcrB. (C) Gold-standard Fourier shell correlation (GS-FSC) curves of apo-*Kp*AcrB. (D) Local EM density map of apo-*Kp*AcrB. Download FIG S1, JPG file, 1.9 MB. Copyright © 2023 Zhang et al. 2023 Zhang et al. https://creativecommons.org/licenses/by/4.0/ This content is distributed under the terms of the Creative Commons Attribution 4.0 International license.

10.1128/mbio.00659-23.4 TABLE S1 *Kp*AcrB cryo-EM data collection and refinement statistics. Download Table S1, PDF file, 0.09 MB. Copyright © 2023 Zhang et al. 2023 Zhang et al. https://creativecommons.org/licenses/by/4.0/ This content is distributed under the terms of the Creative Commons Attribution 4.0 International license.

*Kp*AcrB adopts the overall fold of hydrophobe-amphiphile efflux (HAE)-RND-type proteins and forms a homotrimer (16–21). Each subunit of *Kp*AcrB is mainly composed of a transmembrane domain and a periplasmic domain. The transmembrane domain consists of 12 transmembrane helices (TM1-TM12), whereas the periplasmic domain constitutes six subdomains (PN1, PN2, PC1, PC2, DN, and DC) (Fig. 1A to C).

Subdomains PC1 and PC2 create a periplasmic cleft. This periplasmic cleft can be open or closed and forms an entrance drug-binding site to allow for substrates to enter the pump, as observed in *Ec*AcrB (16, 17, 22), P. aeruginosa MexB (18), A. baumannii AdeB (21, 23), A. baumannii AdeJ (24, 25), Campylobacter jejuni CmeB (20), and Neisseria gonorrhoeae MtrD (19, 26, 27). Presumably, a drug molecule recognized by the entrance drug-binding site (Fig. 1D) will be guided by the flexible loop (F-loop) to arrive at the proximal drug-binding site. It will then pass through the gate loop (G-loop) to reach the distal drug-binding site before being exported by the pump. Therefore, a drug molecule entering the periplasmic cleft would likely be sequentially bound at the proximal site and then distal site before drug extrusion.

The entrance of the *Kp*AcrB periplasmic cleft is surrounded with residues F663, F665, L667, R716, and L827 (Fig. 1D). These entrance residues are conserved with *Ec*AcrB; however, they are not conserved among other HAE-RND pumps. For example, the corresponding entrance residues in the A. baumannii AdeB multidrug efflux pump are M656, V658, W708, and I821, residues that actively participate in anchoring an ethidium molecule at the periplasmic entrance drug-binding site of AdeB (23). Therefore, the exact composition of these entrance residues may play a critical role in substrate specificity and selectivity.

Like A. baumannii AdeB (21, 23), A. baumannii AdeJ (24, 25) and N. gonorrhoeae MtrD (26, 27), a flexible F-loop (^668^PAIVELGT^675^) is found to connect the periplasmic cleft entrance and the proximal multidrug-binding site of *Kp*AcrB. It has been found in *Ec*AcrB that residue I671 (corresponding to I670 in *Kp*AcrB) of the F-loop (Fig. 1D) is critical for drug selectivity (28). As the composition of this F-loop in *Kp*AcrB is identical to that of *Ec*AcrB, it is expected that this isoleucine is also important for the function of *Kp*AcrB.

There are at least 21 amino acids involved in forming the proximal drug-binding site in *Ec*AcrB. Among them, 20 are conserved with those of the *Kp*AcrB pump. In the X-ray structures of rifampicin- and erythromycin (Ery)-bound *Ec*AcrB, these two drugs were found to anchor in the proximal drug-binding site, where residues S79, T91, S134, S135, K292, F617, T624, M662, F664, L674, and D681 specifically contact these drugs (29). Therefore, the corresponding amino acids in *Kp*AcrB are expected to be important for substrate binding (Fig. 1D).

The G-loop of *Kp*AcrB is composed of ^613^GFGFAG^618^, where this conserved G-loop in *Ec*AcrB participates in shuttling substrates from the proximal to distal drug-binding sites (29, 30). Molecular dynamics simulations also depicted that the phenylalanines of this *Ec*AcrB G-loop are critical for facilitating drug transport (30).

The distal drug-binding site of *Ec*AcrB comprises 23 amino acids. Of these 23 residues, 19 of them are identical to those of *Kp*AcrB. Many of these conserved residues are aromatic in nature, including the six phenylalanines F136, F178, F610, F615, F617, and F628 of *Ec*AcrB (corresponding to F136, F178, F609, F614, F616, and F627 of *Kp*AcrB) (Fig. 1D). A mutagenesis study of the N. gonorrhoeae MtrD multidrug efflux pump suggests that mutations on these corresponding phenylalanines significantly reduced resistance of N. gonorrhoeae to different antimicrobials (31). In addition, a hydrophobic patch is found in the distal site of *Ec*AcrB, where the composed hydrophobic residues are critically important in drug binding (30). In *Kp*AcrB, the composition of this distal hydrophobic patch is F178, V277, V611, and F614 (Fig. 1D). These residues are potentially critical for contacting the bound drugs.

Interestingly, the cryo-EM structure of *Kp*AcrB in the absence of drug indicates that this multidrug efflux pump forms an asymmetric trimer in which the three protomers possess distinct conformational states (Fig. 1A to C). Similar to structures of asymmetric *Ec*AcrB (17), P. aeruginosa MexB (18), N. gonorrhoeae MtrD (26, 27), and A. baumannii AdeJ (24, 25), the conformations of the three apo-*Kp*AcrB protomers can be assigned as “extrusion” (conformation of a protomer just after substrate export), “access” (presubstrate-binding form of a protomer with a vacant binding site), and “binding” (conformation of a protomer with a bound substrate) states, respectively (in a clockwise direction as shown Fig. 1B). The assignment of the conformations of these three *Kp*AcrB protomers are shown in Fig. S2 and Table S2. No extra densities were observed within the periplasmic drug-binding sites of these three *Kp*AcrB protomers, suggesting that our cryo-EM structure represents the apo form of *Kp*AcrB (apo-*Kp*AcrB).

10.1128/mbio.00659-23.2 FIG S2 *Kp*AcrB structural state assignment. (A) A protomer of apo-*Kp*AcrB. The red and black boxes indicate the locations of the exit tunnel and proton-relay network, respectively. (B) Distance between residues Q125 and Y757 at the exit site of the “access” (green), “extrusion” (pink), and “binding” (slate) protomers of apo-*Kp*AcrB. Residues Q125 and Y757 are shown as cyan sticks. The distances are measured between the Cα atoms of Q125 and Y757. The cryo-EM densities of these two residues are shown as black mesh. The cryo-EM densities of these residues are shown as black mesh. (C) Conformations of the proton-relay residues of the “access” (green), “extrusion” (pink), and “binding” (slate) protomers of apo-*Kp*AcrB. The cryo-EM densities of these residues are shown as black mesh. Residues (D407, D408, K939, N940, and T977) involved in the proton-relay network are shown as cyan sticks. The hydrogen bonds are highlighted with yellow dotted lines. (D) A protomer of *Kp*AcrB-Ery. The red and black boxes indicate the locations of the exit tunnel and proton-relay network, respectively. (E) Distance between residues Q125 and Y757 at the exit site of the “binding” (slate), “extrusion” (pink), and “access” (green) protomers of *Kp*AcrB-Ery. Residues Q125 and Y757 are shown as cyan sticks. The distance are measured between the Cα atoms of Q125 and Y757. The cryo-EM densities of these two residues are shown as black mesh. (F) Conformations of the proton-relay residues of the “binding” (slate), “extrusion” (pink), and “access” (green) protomers of *Kp*AcrB-Ery. The cryo-EM densities of these residues are shown as black mesh. Residues (D407, D408, K939, N940, and T977) involved in the proton-relay network are shown as cyan sticks. The hydrogen bonds are highlighted with yellow dotted lines. Download FIG S2, JPG file, 1.9 MB. Copyright © 2023 Zhang et al. 2023 Zhang et al. https://creativecommons.org/licenses/by/4.0/ This content is distributed under the terms of the Creative Commons Attribution 4.0 International license.

10.1128/mbio.00659-23.5 TABLE S2 Classification of *Kp*AcrB protomer states. Download Table S2, PDF file, 0.10 MB. Copyright © 2023 Zhang et al. 2023 Zhang et al. https://creativecommons.org/licenses/by/4.0/ This content is distributed under the terms of the Creative Commons Attribution 4.0 International license.

### Structure of K. pneumoniae AcrB in the presence of erythromycin.

**FIG 2:**
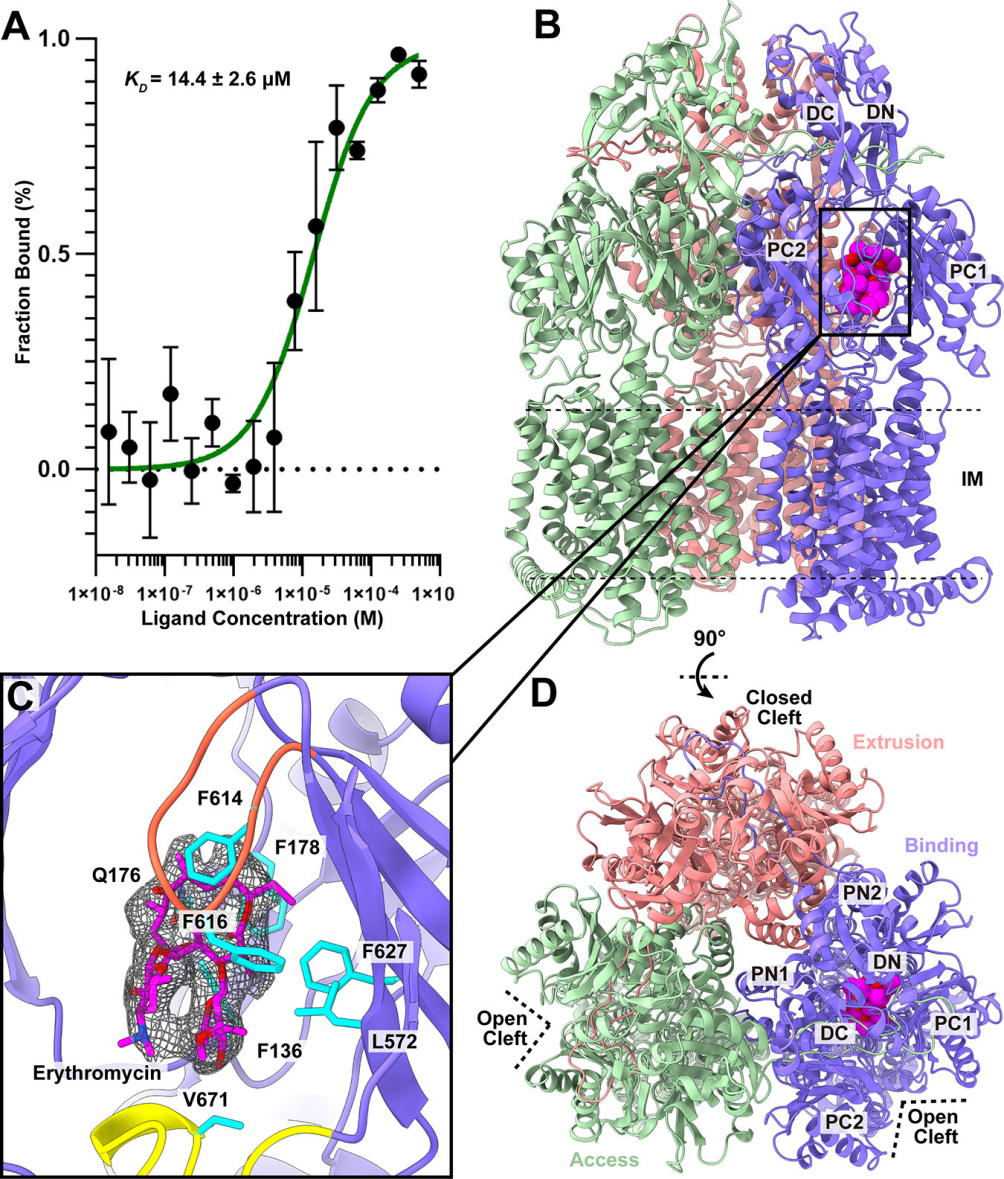
Cryo-EM structure of *Kp*AcrB-Ery. (A) Interaction between *Kp*AcrB and Ery. The binding curve obtained from microscale thermophoresis (MST) depicts that the equilibrium dissociation constant (*K_D_*) for *Kp*AcrB and Ery interaction is 14.4 ± 2.6 μM. (B) Ribbon diagram of the structure of the side view (viewed in the membrane plane) of the *Kp*AcrB trimer. The “extrusion,” “binding,” and “access” protomers are colored pink, slate, and green, respectively. The bound Ery molecule in the “binding” protomer is shown as magenta balls. The inner membrane (IM) is highlighted with dotted lines. (C) The Ery-binding site. The residues involved in Ery binding are shown as cyan sticks. The bound Ery molecule is in magenta sticks. Cryo-EM density of bound Ery is in black mesh. The F- and G-loops are colored yellow and orange. (D) Ribbon diagram of the structure of the top view (viewed from the extracellular space) of the *Kp*AcrB trimer. The “extrusion,” “binding,” and “access” protomers are colored pink, slate, and green, respectively. In panels A and C, each protomer of *Kp*AcrB contains 12 transmembrane helices (TM1 to TM12) and six periplasmic subdomains (PN1, PN2, PC1, PC2, DN, and DC).

To elucidate how *Kp*AcrB recognizes drugs, we chose the Ery (C_37_H_67_NO_13_) macrolide for our ligand-bound structural studies. Ery contains 37 carbons, 1 nitrogen, 13 oxygens, and 67 hydrogen with a molecular weight of 733.9 g/mol. We rationalized that the relatively large size of this antibiotic should allow us to unambiguously identify the location of this bound drug within the pump. We first quantified the binding affinity for *Kp*AcrB and Ery interaction using the technique of microscale thermophoresis (MST) (32). The MST analysis indicates that the equilibrium dissociation constant (*K_D_*) for *Kp*AcrB and Ery interaction is 14.4 ± 2.6 μM (Fig. 2A). This *K_D_* value is in good agreement with those for *Ec*AcrB, where *Ec*AcrB binds different substrates, including rhodamine 6G, ethidium, proflavine and ciprofloxacin, with *K_D_* values ranging from 5.5 to 74.1 μM (33).

After confirming that *Kp*AcrB specifically interacts with Ery, we incubated 10 μM *Kp*AcrB-nanodisc sample with 500 μM Ery for 2 h to form the *Kp*AcrB-Ery complex. We then solved the cryo-EM structure of this complex to a resolution of 2.96 Å (Fig. 2B to D; Fig. S3; Table S1). The overall structure of *Kp*AcrB-Ery is very similar to that of apo-*Kp*AcrB. Superimposition of the apo-*Kp*AcrB and *Kp*AcrB-Ery trimers gives rise to an overall root-mean-square deviation (r.m.s.d.) of 0.36 Å. In comparison with the structure of apo-*Kp*AcrB, the conformation of *Kp*AcrB-Ery is distinct in that the three protomers are in the forms of “extrusion,” “binding,” and “access,” respectively (in a clockwise direction as shown Fig. 2D) instead of “extrusion,” “access,” and “binding” conformations (in a clockwise direction) as shown in the apo-*Kp*AcrB trimer structure. The assignment of conformational states of these *Kp*AcrB protomers are shown in Fig. S2 and Table S2.

10.1128/mbio.00659-23.3 FIG S3 *Kp*AcrB-Ery data processing. (A) Data processing workflow of *Kp*AcrB-Ery. Side and top views of the *Kp*AcrB-Ery density maps are shown. (B) Representative two-dimensional classes *Kp*AcrB-Ery. (C) Gold-standard Fourier shell correlation (GS-FSC) curves of *Kp*AcrB-Ery. (D) Local EM density map of *Kp*AcrB-Ery. Download FIG S3, JPG file, 1.8 MB. Copyright © 2023 Zhang et al. 2023 Zhang et al. https://creativecommons.org/licenses/by/4.0/ This content is distributed under the terms of the Creative Commons Attribution 4.0 International license.

Within the “binding” protomer of *Kp*AcrB-Ery, we discovered a large extra density corresponding to the bound Ery macrolide (Fig. 2C). This Ery molecule is located deep inside the distal drug-binding site of the *Kp*AcrB pump. No extra densities were seen in the periplasmic clefts of the “access” and “extrusion” protomers, suggesting that only the “binding” protomer is occupied by Ery. It is interesting to note that the crystal structure of *Ec*AcrB-Ery depicts a relatively different binding mode for Ery, where this macrolide was anchored in the proximal drug-binding site and not the distal site of the *Ec*AcrB pump (29).

Within the deep distal binding pocket of *Kp*AcrB, the binding of Ery is quite extensive. There are six aromatic and hydrophobic residues, including F136, F178, Y327, F614, F616, and L667, involved in anchoring this drug. Notably, the distal hydrophobic patch residues F178 and F614 contribute to bind Ery. Interestingly, this drug-binding mode is very similar to that found in the N. gonorrhoeae MtrD_CR103_ multidrug efflux pump, where the Ery macrolide was also bound within the distal drug-binding pocket of MtrD_CR103_ (26).

*Kp*AcrB uses the proton-motive force (PMF) to energize the drug efflux process (14). In the transmembrane region of *Ec*AcrB, the conserved residues D407, D408, K940, N941, and T978 create a proton-relay network for energy coupling (34, 35). The corresponding residues in *Kp*AcrB are D407, D408, K939, N940, and T977 (Fig. S2). These conserved residues are also found in the A. baumannii AdeB (21, 23), A. baumannii AdeJ (24, 25), C. jejuni CemB (20), E. coli AcrD (36), and N. gonorrhoeae MtrD (26, 27) efflux pumps to participate in the proton-relay networks. Therefore, these *Kp*AcrB residues are likely responsible for the translocation of protons from the periplasm to the cytoplasm and generating the PMF necessary for extruding drugs from the cell. The high-quality densities of our cryo-EM maps unambiguously depict the conformations of the side chains of these conserved amino acids. This, in turn, allows us to predict and delineate a possible mechanism concerning the process of proton transfer within this proton-relay network, as well as how the influx of protons and the efflux of drugs are coupled together. The conformations of these side chains also allow us to identify different conformational states of the pump.

### Docking of substrates into the multidrug-binding sites.

**FIG 3:**
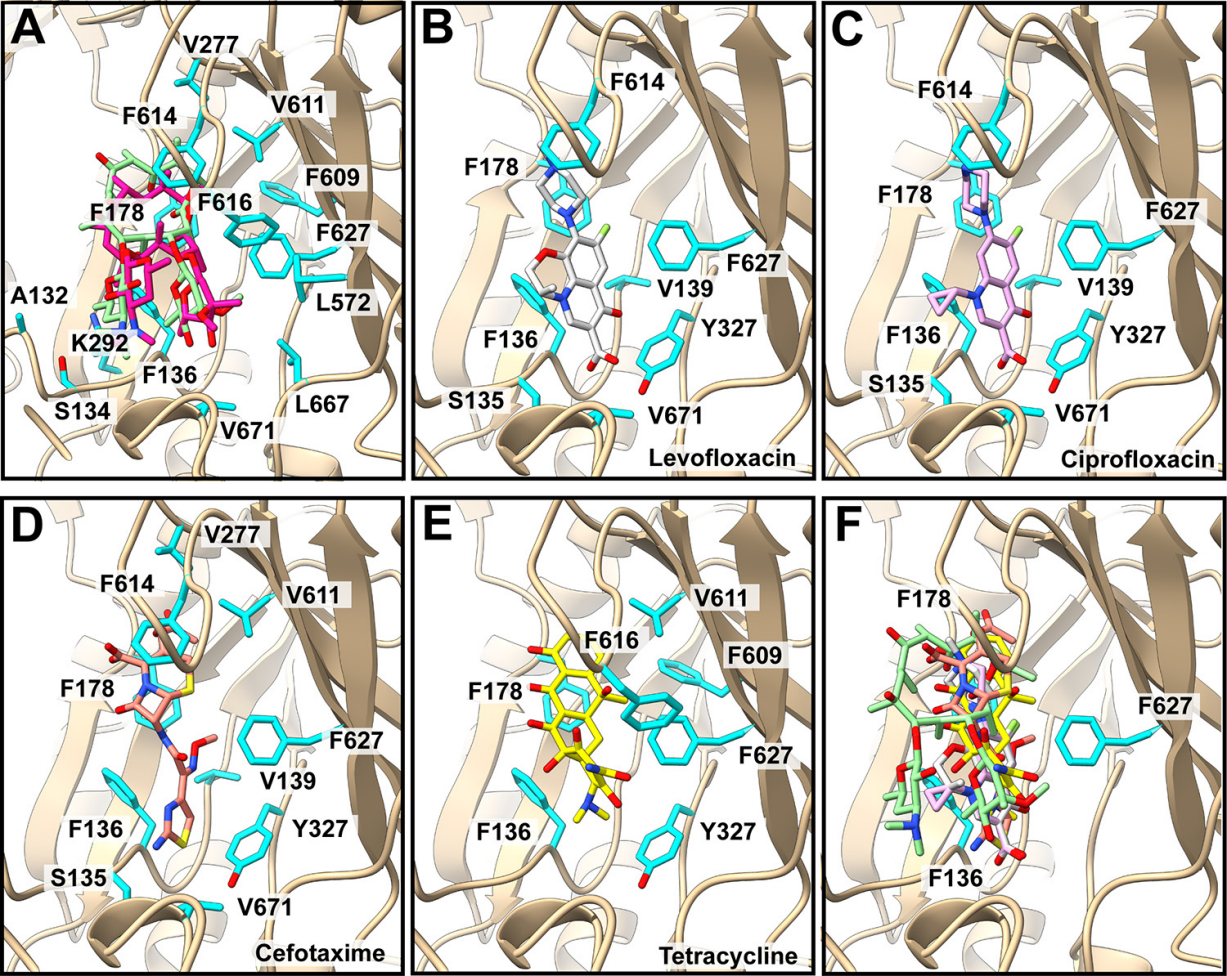
Docking of antibiotics to the structure of *Kp*AcrB. (A) Predicted Ery-binding site. The docked Ery molecule is shown as green sticks. The bound Ery antibiotic identified from our cryo-EM structure is also included and is shown as magenta sticks. Residues predicted to be responsible for Ery binding are in cyan sticks. (B) Predicted Lev-binding site. The docked Lev molecule is shown as gray sticks. Residues predicted to be responsible for Lev binding are in cyan sticks. (C) Predicted Cip-binding site. The docked Cip molecule is shown as pink sticks. Residues predicted to be responsible for Cip binding are cyan sticks. (D) Predicted Cef-binding site. The docked Cef molecule is shown as orange sticks. The residues predicted to be responsible for Cef binding are in cyan sticks. (E) Predicted Tet-binding site. The docked Tet molecule is shown as yellow sticks. The residues predicted to be responsible for Tet binding are in cyan sticks. (F) Composite figure showing the locations of predicted bound antibiotics at the distal multidrug-binding site (green, Ery; gray, Lev; pink, Cip; orange, Cef; yellow, Tet). The three residues (F136, F178, and F627) predicted to participate in the binding of all of these five antibiotics are in cyan sticks.

The cryo-EM structures of *Kp*AcrB have allowed us to understand specific interactions between this pump and the Ery macrolide. We decided to predict how *Kp*AcrB is capable of recognizing and accommodating different classes of antibiotics using the program AutoDock Vina (37). We chose to dock levofloxacin (Lev), ciprofloxacin (Cip), cefotaxime (Cef), and tetracycline (Tet), as *Kp*AcrB can mediate a high level of resistance to these drugs (14). To ensure the confidence and reliability of these docking calculations, we first docked Ery into the distal multidrug-binding site of *Kp*AcrB and observed that Ery was bound at the same location as seen in the cryo-EM structure of *Kp*AcrB-Ery (Fig. 3A). We then studied the interactions of *Kp*AcrB with Lev, Cip, Cef, and Tet using the same approach. The docking predictions suggest that *Kp*AcrB specifically contacts and houses these drugs in the distal multidrug-binding site. The locations of these bound compounds also overlap within this site (Fig. 3B to F). However, it appears that *Kp*AcrB may utilize slightly different subsets of residues to interact with these different drugs based on the docking results (Table S3). The predicted binding affinities for these antibiotics are −8.2 kcal/mol (for Ery), −8.1 kcal/mol (for Lev), −7.8 kcal/mol (for Cip), −6.3 kcal/mol (for Cef), and −8.1 kcal/mol (for Tet) (Table S3). The docking calculations also suggest that F136, F178, and F627 (highlighted with red in Table S3) are commonly used to for binding these five antibiotics. These three phenylalanines could be very important for recognizing multiple drugs.

10.1128/mbio.00659-23.6 TABLE S3 Docking of antibiotics to *Kp*AcrB. Download Table S3, PDF file, 0.09 MB. Copyright © 2023 Zhang et al. 2023 Zhang et al. https://creativecommons.org/licenses/by/4.0/ This content is distributed under the terms of the Creative Commons Attribution 4.0 International license.

## DISCUSSION

The emergence of multidrug-resistant K. pneumoniae significantly challenges our efforts to treat and combat these infectious bacterial diseases. This, coupled with the current COVID-19 global health emergency, further pressurizes antimicrobial stewardship activities, as the pandemic provides a perfect breeding ground for opportunistic pathogens such as K. pneumoniae to establish coinfections in COVID-19 patients. Recently, the Centers for Disease Control and Prevention (CDC) has listed KPC-producing K. pneumoniae in the highest antimicrobial resistance threat category (38). Infections caused by this bacterium are difficult to treat and often associated with a very low survival rate. There is also an increasing trend of resistance to carbapenems, tigecycline and even the last resort drug colistin (39, 40) that severely limits the choice of therapies. This is, in a significant part, due to the presence of highly efficient multidrug efflux pumps that these bacteria carry to mediate antimicrobial resistance.

To initiate a research effort for structure-based drug design to combat infections caused by K. pneumoniae, we defined cryo-EM structures of the *Kp*AcrB multidrug efflux pump, in both the absence and the presence of the Ery macrolide. *Kp*AcrB is the most predominant and clinically important multidrug efflux pump in K. pneumoniae. This pump relies on the PMF and functions via a drug/proton antiport mechanism. Coupled with the export of drug molecules toward the periplasm, protons need to be imported into the cytoplasm to energize this efflux process. Our cryo-EM maps unambiguously depict conformational changes of the side chains of residues D407, D408, K939, N940, and T977. These important residues are conserved among the HAE-RND efflux pumps and create the proton-relay network within the transmembrane helices. It has been found that a conserved lysine residue is critically important for proton transfer across the proton-relay network, where it participates as a proton sweeper to guide this process across the cytoplasmic membrane. In our cryo-EM structures, we observed that the conformation of K939 is quite distinct in protomers with different conformational states. This observation is in good agreement with what is seen in the N. gonorrhoeae MtrD (26, 27), A. baumannii AdeB (21, 23), and A. baumannii AdeJ (24, 25) multidrug efflux pumps. Therefore, the proton transfer mechanism found in *Kp*AcrB may generally apply to inner membrane RND pumps in a myriad of bacterial organisms.

RND pumps often show a broad range of substrate specificity and play an important role in mediating intrinsic antibiotic resistance by directly extruding these drugs out of Gram-negative bacterial cells. However, it has been quite challenging to quantify drug efflux rates via these pumps mainly because these pumps need form a tripartite complex, spanning both the inner and the outer membranes, in order to export drugs. Using intact E. coli cells harboring the complete E. coli AcrAB-TolC multiprotein complex, the efflux rates of penicillins and cephalosporins from this complex have been determined. These experiments suggested that the *V*_max_ (maximum velocity) values are between 0.35 and 1.1 nmol/mg/s for efflux of penicillins (41), whereas these values are between 0.023 and 0.37 nmol/mg/s for efflux of cephalosporins (42).

Based upon the cryo-EM structures, we observed Ery to be bound deeply inside the periplasmic cleft of its distal drug-binding site. This pump mainly relies on aromatic residues, such as F136, F178, Y327, F614, F616, and F627, to recognize and anchor the bound Ery molecule. How Ery binds in *Kp*AcrB is very distinct from the interactions observed in *Ec*AcrB (29) but quite similar to the mode of Ery binding in the N. gonorrhoeae MtrD pump (26), where MtrD was found to house Ery in the distal drug-binding site with aromatic residues F136, F176, Y325, F568, F610, F612, and F625 responsible for binding.

Macrolides typically target the large 50S ribosomal subunit of bacterial 70S ribosomes. These drugs inhibit ribosome translocation along the mRNA chain, resulting in cessation of bacterial protein synthesis. In a structural study of the S. aureus 70S ribosome (43), it was found that the large 50S ribosomal subunit utilizes aromatic rings of eight different nucleotides to secure the binding of the Ery macrolide. This binding mode is indeed very similar to that found in the *Kp*AcrB multidrug efflux pump, in which six aromatic residues are involved in performing aromatic stacking interactions with the bound Ery drug.

In addition to antibiotic resistance, it has been demonstrated that *Kp*AcrB is capable of mediating resistance to a host of antimicrobial peptides (14). How the *Kp*AcrB pump interacts with these peptides is not known. Recently, a cryo-EM structure of the N. gonorrhoeae MtrD multidrug efflux pump bound by a linear human cationic antimicrobial peptide derived from human LL-37 has been reported (27). This article also included two additional cryo-EM structures of MtrD bound by a novel nonantimicrobial cyclic cationic antimicrobial peptide and a colistin antimicrobial peptide (27). Each peptide was found to bind within the periplasmic cleft region of MtrD, in which the entrance, proximal, and distal drug-binding sites are involved in anchoring these peptides. There is a good chance that *Kp*AcrB may utilizes a similar mechanism to recognize these host antimicrobial peptides.

Our computational docking results predict that *Kp*AcrB can specifically bind and house a variety of drugs, including Lev, Cip, Cef, and Tet, at the distal multidrug-binding site. A signature for a multidrug-binding protein is that it is able to use slightly different subsets of residues within a large substrate-binding pocket to accommodate a variety of small compounds. Docking data suggest that *Kp*AcrB indeed utilizes slightly different residues to anchor structurally dissimilar drugs (Table S3). In most cases, *Kp*AcrB employs aromatic and hydrophobic residues, such as F136, F178, F614, F627, and V671, to contact structurally distinct drugs. Thus, drug recognition in *Kp*AcrB appears to be mainly governed by hydrophobic interactions.

Previously, we solved cryo-EM structures of the A. baumannii AdeJ multidrug efflux pump bound with the tetracycline class of drugs, eravacycline and TP-6076, respectively (24, 25). We observed that these two drugs were bound within the distal drug-binding site of AdeJ. The AdeJ pump utilized several aromatic residues, including F136, F178, F277, Y327, F611, F613, F616, F618, and F629, to anchor these drugs (24, 25). The mode of binding for these two tetracycline drugs by AdeJ is very similar to that found in the docking calculation for Tet in this study, in which the *Kp*AcrB pump uses the aromatic residues F136, F178, Y327, F609, V611, F616, and F627 to contact Tet. Further structural studies of this pump with other antibiotics are needed in order to fully understand the detailed mechanism of multidrug recognition utilized by this efflux pump. Our studies will ultimately inform an era in structure-guided drug design to combat multidrug resistance in these Gram-negative pathogens.

## MATERIALS AND METHODS

### Expression and purification of *Kp*AcrB.

The K. pneumoniae AcrB multidrug efflux pump was cloned into the pET15bΩ*KpacrB* expression vector in frame with a 6×His tag at the N terminus. The plasmid was transfected into E. coli BL21(DE3)Δ*acrB* cells, which harbor a deletion in the chromosomal *acrB* gene of E. coli, for overproduction of the *Kp*AcrB membrane protein. The cells were grown in 6 liters of Luria-Bertani (LB) medium supplemented with 100 μg/mL ampicillin at 37°C. When the optical density at 600 nm (OD_600 nm_) reached 0.5, the expression of *Kp*AcrB was induced with 0.2 mM isopropyl-β-d-thiogalactopyranoside (IPTG). The cells were then harvested within 4 h of induction. The collected bacterial cells were resuspended in low-salt buffer (100 mM sodium phosphate, pH 7.2, 10% glycerol, 1 mM EDTA, and 1 mM phenylmethanesulfonyl fluoride [PMSF]) and disrupted with a French pressure cell. The membrane fraction was collected and washed twice with high-salt buffer (20 mM sodium phosphate, pH 7.2, 2 M KCl, 10% glycerol, 1 mM EDTA, and 1 mM PMSF) and once with final buffer (20 mM Na-HEPES, pH 7.5, and 1 mM PMSF). The membrane protein was then solubilized in 2% (wt/vol) *n*-dodecyl-β-d-maltoside (DDM). Insoluble material was removed by ultracentrifugation at 100,000 × *g*. The extracted protein was then purified with a Ni^2+^-affinity column. The purity of the *Kp*AcrB protein (>95%) was judged using SDS-PAGE stained with Coomassie brilliant blue. The purified protein was dialyzed against 20 mM Na-HEPES (pH 7.5) and concentrated to 7 mg/mL (60 μM) in a buffer containing 20 mM Na-HEPES (pH 7.5) and 0.05% DDM.

### Nanodisc preparation.

To assemble *Kp*AcrB into nanodiscs, a mixture containing 20 μM *Kp*AcrB, 45 μM MSP (1E3D1) and 930 μM E. coli total extract lipid was incubated for 15 min at room temperature. A total of 0.8 mg/mL prewashed Bio-beads (Bio-Rad) was added to remove the DDM detergent. The resultant mixture was incubated for 1 h on ice followed by overnight incubation at 4°C. The protein-nanodisc solution was filtered through 0.22-μm nitrocellulose filter tubes to remove the Bio-beads. To separate free nanodiscs from *Kp*AcrB-loaded nanodiscs, the filtered protein-nanodisc solution was purified using a Superose 6 column (GE Healthcare) equilibrated with 20 mM Tris-HCl, pH 7.5, and 100 mM NaCl. Fractions corresponding to the size of the trimeric *Kp*AcrB-nanodisc complex were collected for cryo-EM sample preparation. 

### Cryo-EM sample preparation.

For imaging apo-*Kp*AcrB, a 10 μM *Kp*AcrB-nanodisc sample was directly applied to glow-discharged holey carbon grids (Quantifoil Cu R1.2/1.3, 300 mesh), blotted for 18 s, and then plunge-frozen in liquid ethane using a Vitrobot (Thermo Fisher). For imaging *Kp*AcrB-Ery, a 10 μM *Kp*AcrB-nanodisc sample was incubated with 500 μM Ery for 2 h to form the *Kp*AcrB-Ery complex. The sample was then applied to glow-discharged holey carbon grids (Quantifoil Cu R1.2/1.3, 300 mesh), blotted for 18 s, and then plunge-frozen in liquid ethane using a Vitrobot (Thermo Fisher). All grids were then transferred into cartridges prior to data collection.

### Data collection.

For the apo-*Kp*AcrB sample, the images were collected in super-resolution mode at 81 K magnification on a Titan Krios equipped with a K3 direct electron detector (Gatan). The physical pixel size was 1.07 Å/pix (super-resolution of 0.535 Å/pix). Each micrograph was exposed to a total dose of 35.5 e-/Å^2^ for 3.5 s, and 37 frames were captured using SerialEM (44). For the *Kp*AcrB-Ery sample, each micrograph was collected over 38 frames with a total dose of 37.7 e-/Å^2^ over 3.5 s using SerialEM (44).

### Data processing.

For apo-*Kp*AcrB, the super-resolution image stack was aligned and binned by 2 using patch motion. The contrast transfer function (CTF) was estimated using patch CTF in cryoSPARC (45). A procedure for blob picker followed by two-dimensional (2D) classification were applied to generate templates for automated template picking. Initially, 731,684 particles were selected after autopicking in cryoSPARC (45). Several iterative rounds of 2D classifications followed by *ab initio* and heterogeneous three-dimensional (3D) classifications were performed to remove false picks, and classes with unclear features, ice contamination, or carbonA single round of nonuniform refinement followed by local refinement with nonuniform sampling resulted in 2.82 Å resolution cryo-EM maps for apo-*Kp*AcrB based on the gold-standard Fourier shell correlation (FSC 0.143) (Fig. S1).

For *Kp*AcrB-Ery, the same procedure was used to generate templates for automated template picking. Initially, 1,459,014 particles were selected after autopicking in cryoSPARC (45). Several iterative rounds of 2D classifications, *ab initio* and heterogeneous 3D classifications were performed to remove false picks and classes with unclear features. Nonuniform refinement followed by local refinement with nonuniform sampling resulted in 2.96 Å resolution cryo-EM maps for *Kp*AcrB-Ery based on the gold-standard Fourier shell correlation (FSC 0.143) (Fig. S3).

### Model building and refinement.

Model buildings of were based on the cryo-EM maps, respectively. A predicted *Kp*AcrB structure using AlphaFold (46) was used and fitted into the corresponding density maps using Chimera (47). The subsequent model rebuilding was performed using Coot (48). Structural refinements were performed using the phenix.real_space_refine program (49) from the PHENIX suite (50). The final atomic model was evaluated using MolProbity (51). The statistics associated with data collection, 3D reconstruction, and model refinement are included in Table S1.

### Microscale thermophoresis assay.

The MST experiment was performed using a Monolith NT.Labelfree instrument (NanoTemper Technologies GmbH, Munich, Germany) coupled with premium standard capillaries (NanoTemper Technologies, Munich, Germany). A 16-point dilution series of Ery was made by dissolving Ery in 20 mM Na-HEPES (pH 7.5) and 0.02% DDM. Each Ery solution was then mixed with the *Kp*AcrB protein solution in 20 mM Na-HEPES (pH 7.5) and 0.02% DDM. After 15-min incubation, these samples were filled into 16 different premium standard capillaries, respectively. The final concentration of *Kp*AcrB in each capillary was 500 nM. The final concentration of Ery ranged between 15 nM and 500 μM. Measurements were taken with excitation wavelength at 280 nm and emission wavelength at 360 nm. For each measurement, MST signal was recorded within 25 s, and laser-on time was set at 20 s and laser-off time was set at 5 s. The experiment was repeated three times to ensure reproducibility.

### Molecular modeling.

The program AutoDock Vina (37) was used to predicted the drug-binding modes of five *Kp*AcrB drugs, including Ery, Lev, Cip, Cef, and Tet. The “binding” protomer of the *Kp*AcrB-Ery structure (with the Ery molecule removed) was used for dockings. The protein was set as a rigid structure, whereas the conformation of each antibiotic molecule was optimized via all modeling and docking procedures. For each drug, the results were ranked on the basis of predicted free binding energy, and the one with the highest binding affinity was recorded (Table S3).

### Data availability.

The atomic coordinates and EM maps of apo-*Kp*AcrB and *Kp*AcrB-Ery have been deposited with PDB accession codes 8FFK and 8FFS and EMDB accession codes EMD-29045 and EMD-29055.
