# Supplementary figures and images for: Cryo-EM Structures of the Klebsiella pneumoniae AcrB Multidrug Efflux Pump
Source: mBio. 2023 Apr 17;14(3):e00659-23. doi: 10.1128/mbio.00659-23 (PMC10294659; doi:10.1128/mbio.00659-23)

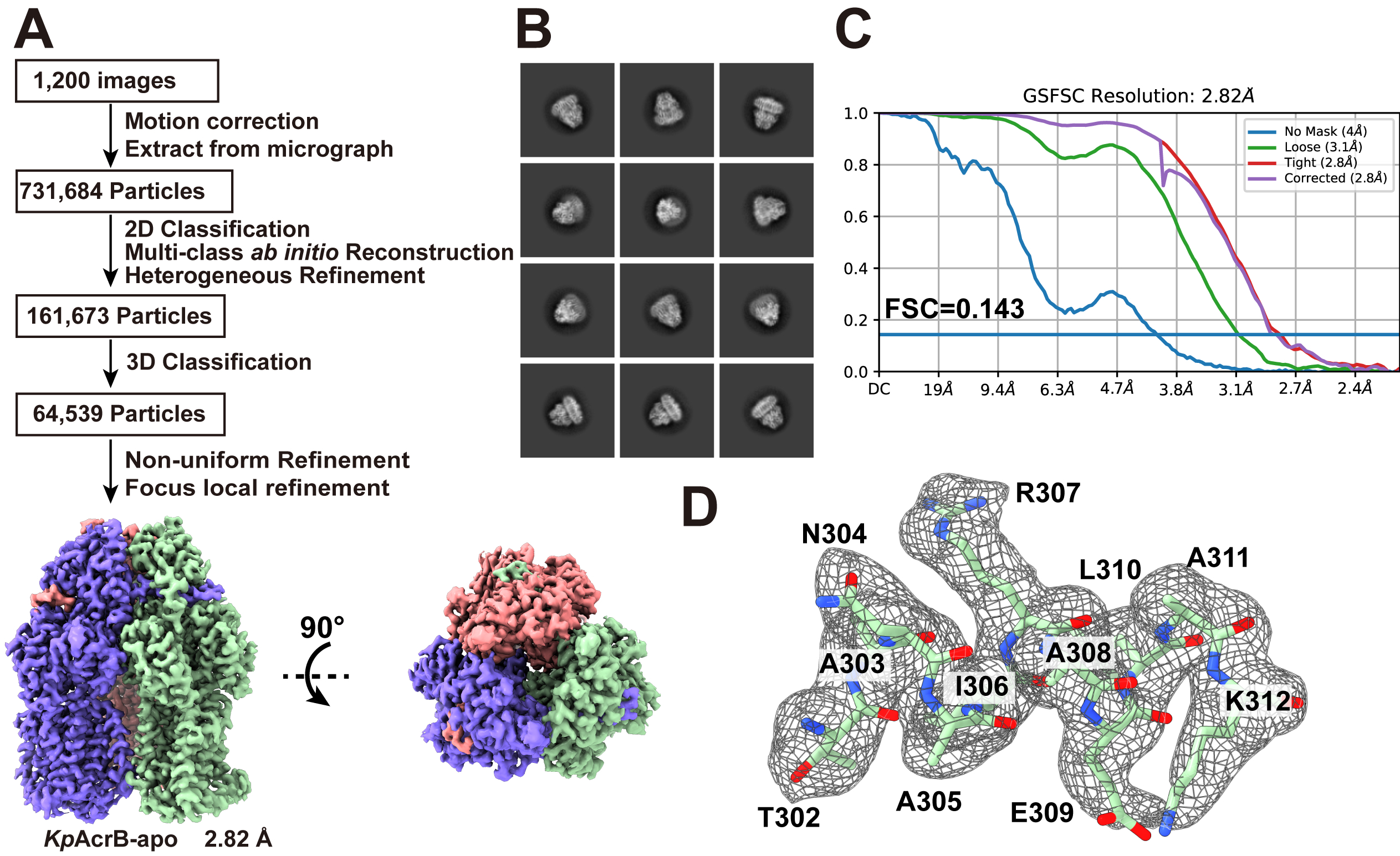

Supplement: FIG S1 [file mbio.00659-23-s0001.jpg]

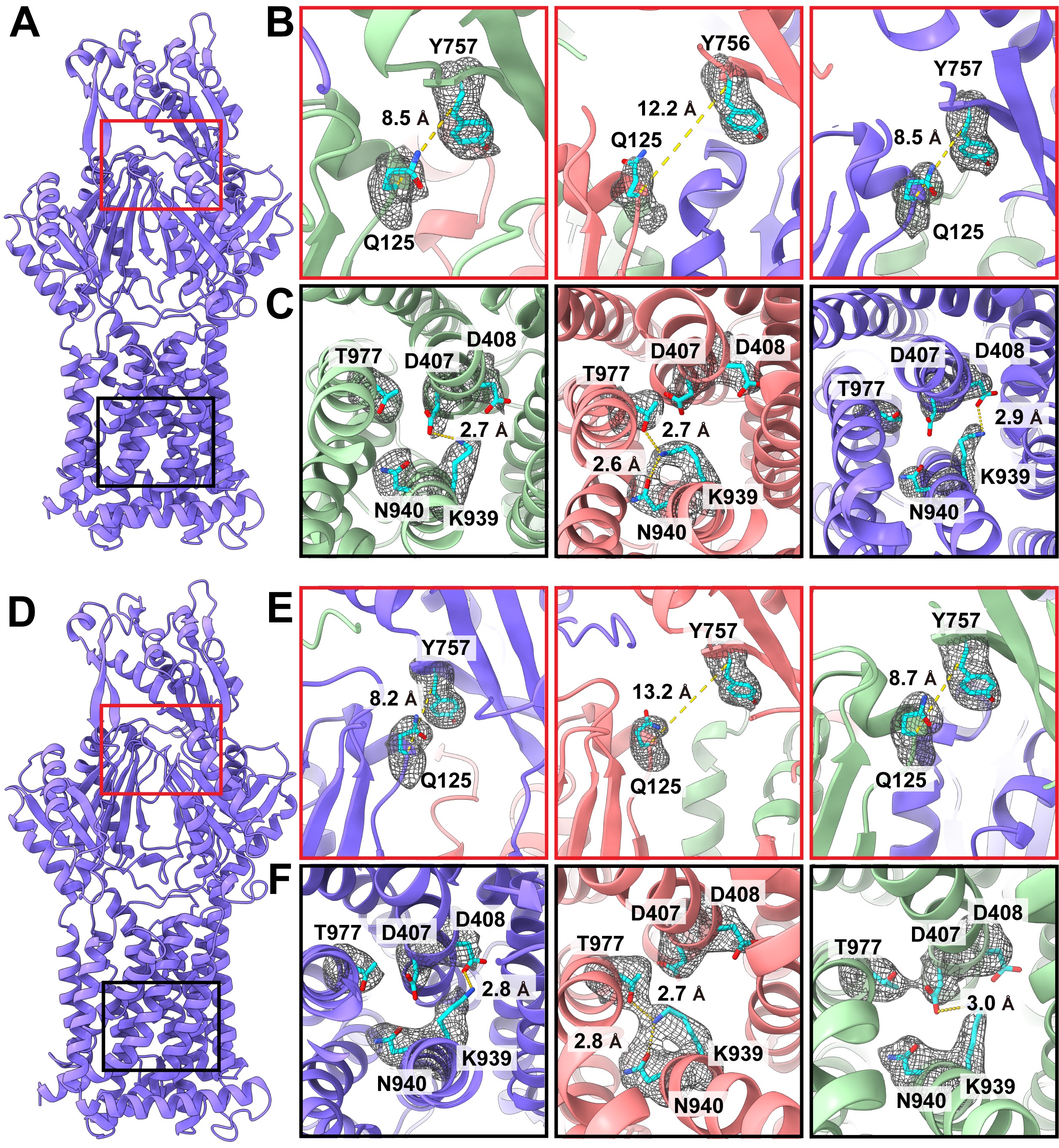

Supplement: FIG S2 [file mbio.00659-23-s0002.jpg]

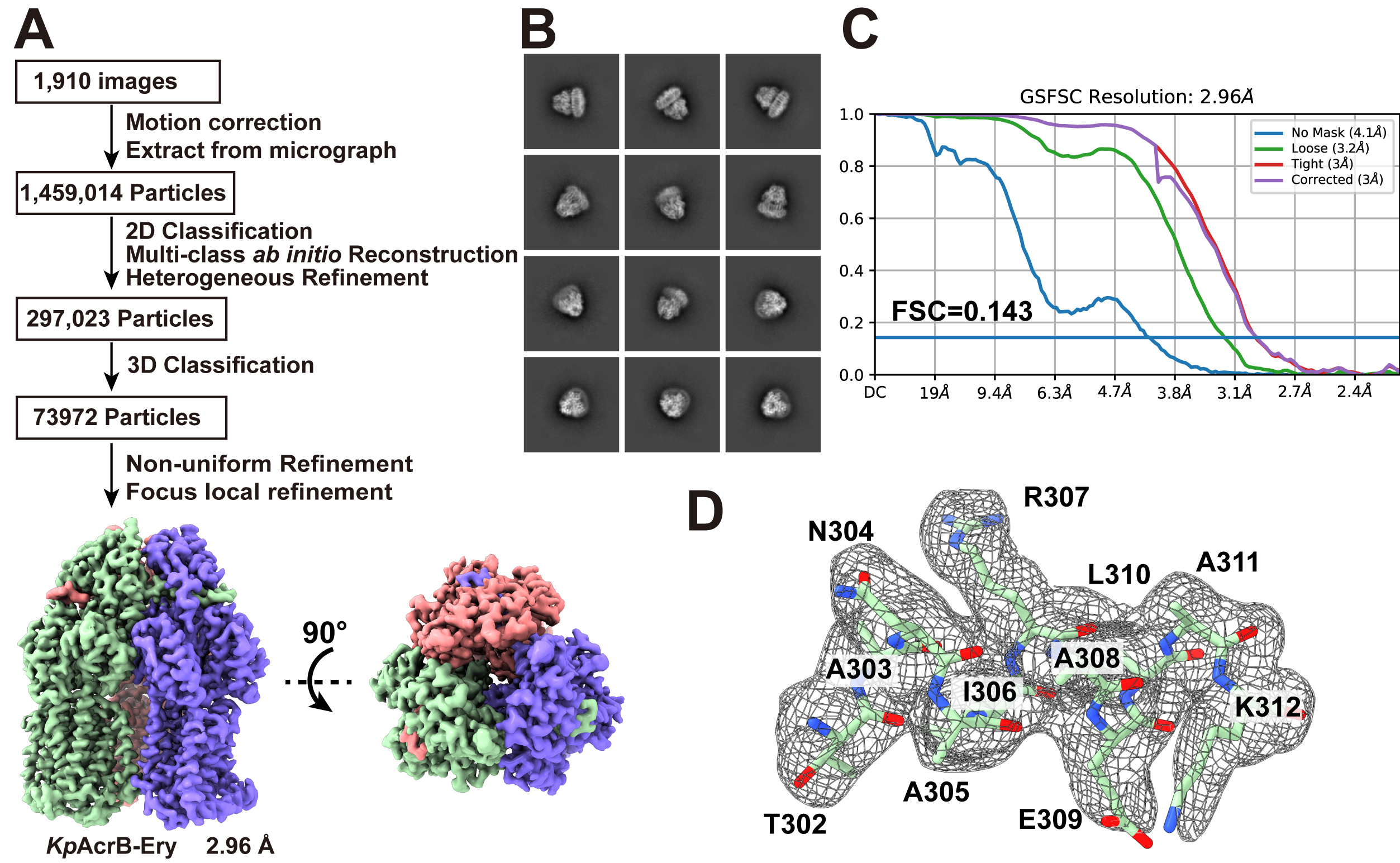

Supplement: FIG S3 [file mbio.00659-23-s0003.jpg]
