# Supplementary material for: Cryo-EM Structures of the Klebsiella pneumoniae AcrB Multidrug Efflux Pump
Source: mBio. 2023 Apr 17;14(3):e00659-23. doi: 10.1128/mbio.00659-23 (PMC10294659; doi:10.1128/mbio.00659-23)
Supplement: TABLE S1 [file mbio.00659-23-s0004.pdf]

Table S1. ***Kp*AcrB cryo-EM data collection and refinement statistics.**

**Data collection**	**Apo-*Kp*AcrB**	***Kp*AcrB-Ery**
Magnification	81,000	81,000
Voltage (kV)	300	300
Electron Microscope	Krios-GIF-K3	Krios-GIF-K3
Defocus (um)	-0.8 to -1.5	-0.8 to -1.5
Energy filter width (eV)	20	20
Pixel size (Å)	1.07 (0.535)	1.07 (0.535)
Total dose (e^{-}/ $Å^{2}$)	35.5	37.7
Number of frames	37	38
Number of micrographs	1,200	1,910
Initial particle images (no.)	731,684	1,459,014
**Refinement**		
Total Particles (no.)	64,539	73,972
GS-FSC Resolution (0.143, Å)[a]	2.82	2.96
Model composition		
Chains	3	3
Protein residues	3,098	3,099
Ligand	0	1
r.m.s.d.		
Bond lengths (Å)	0.004	0.006
Bond angles (°)	0.539	0.736
**Validation**		
MolProbity score	1.66	1.69
Clash score	7.35	7.29
Ramachandran plot		
Favored (%)	98.97	98.51
Allowed (%)	1.03	1.49
Disallowed (%)	0.00	0.00
CC Mask	0.76	0.86

[a]Gold-Standard Fourier-Shell Correlation
