# Supplementary material for: Cryo-EM Structures of the Klebsiella pneumoniae AcrB Multidrug Efflux Pump
Source: mBio. 2023 Apr 17;14(3):e00659-23. doi: 10.1128/mbio.00659-23 (PMC10294659; doi:10.1128/mbio.00659-23)
Supplement: TABLE S2 [file mbio.00659-23-s0005.pdf]

Table S2. Classification of *Kp*AcrB protomer states.

Protomer	Cleft State	Exit site distance, Q125 to Y757	Hydrogen-bonded distance, K939 to				Protomer Assignment
			D407 (Å)	D408 (Å)	N940 (Å)	T977 (Å)	
Apo-*Kp*AcrB, A	Open	8.5	2.7	-	-	-	Access
Apo-*Kp*AcrB, B	Closed	12.2	-	-	2.6	2.7	Extrusion
Apo-*Kp*AcrB, C	Open	8.5	-	2.9	-	-	Binding
*Kp*AcrB-Ery, A	Open	8.2	-	2.8	-	-	Binding
*Kp*AcrB-Ery, B	Closed	13.2	-	-	2.8	2.7	Extrusion
*Kp*AcrB-Ery, C	Open	8.7	3.0	-	-	-	Access

*Kp*AcrB protomers were defined using three criteria; state of the periplasmic cleft (open or closed), measurement of the exit site (distance between Cα atoms of Q125 and Y757) and hydrogen bond distances of the PTC (K939 to D407, D408, N940 and T977). Using these results, protomers were assigned as either access, binding or extrusion.
