# Supplementary material for: Cryo-EM Structures of the Klebsiella pneumoniae AcrB Multidrug Efflux Pump
Source: mBio. 2023 Apr 17;14(3):e00659-23. doi: 10.1128/mbio.00659-23 (PMC10294659; doi:10.1128/mbio.00659-23)
Supplement: TABLE S3 [file mbio.00659-23-s0006.pdf]

Table S3. Docking of antibiotics to *Kp*AcrB.

**Drug binding affinity**	
**Antibiotics**	**Binding Affinity (kcal/mol)**
Erythromycin	-8.2
Levofloxacin	-8.1
Ciprofloxacin	-7.8
Cefotaxime	-6.3
Tetracycline	-8.1

Residues responsible for drug binding

**Erythromycin (cryo-EM)**	**Erythromycin (Docking)**	**Levofloxacin (Docking)**	**Ciprofloxacin (Docking)**	**Cefotaxime (Docking)**	**Tetracycline (Docking)**
	A132				
	S134				
		S135	S135	S135	
F136	F136	F136	F136	F136	F136
		V139	V139	V139	
Q176					
F178	F178	F178	F178	F178	F178
	V277			V277	
	K292				
		Y327	Y327	Y327	Y327
L572	L572				
	F609				F609
	V611			V611	V611
F614	F614	F614	F614	F614	
F616	F616				F616
F627	F627	F627	F627	F627	F627
	L667				
V671	V671	V671	V671	V671	
